# Transcriptomic insights into cultivation-driven virulence in *Aeromonas* spp.: a new approach to optimizing autogenous vaccines in aquatic veterinary medicine

**DOI:** 10.1186/s13567-025-01692-9

**Published:** 2026-01-24

**Authors:** Dongqing Zhao, Konrad Wojnarowski, Paulina Cholewińska, Tomasz Strzała, Peter Steinbauer, Dušan Palić

**Affiliations:** 1 Chair for Fish Diseases and Fisheries Biology, Faculty of Veterinary Medicine, Ludwig-Maximilians-University Munich, 80539 Munich, Germany; 2 Department of Genetics, Faculty of Biology and Animal Science, Wrocław University of Environmental and Life Sciences, Wrocław, Poland; 3 Tiergesundheitsdienst Bayern e.V., Poing, Bayern Germany

**Keywords:** NGS, autogenous vaccine development, fish, shrimp, aquaculture, disease prevention

## Abstract

Autogenous vaccines are a critical tool in aquaculture for managing bacterial diseases when commercial vaccines are unavailable or ineffective. To improve vaccine efficacy, this study explored how different cultivation conditions influence virulence gene expression in two major fish pathogens, *Aeromonas salmonicida* subsp. *salmonicida* and *Aeromonas hydrophila*. Isolates were cultured in nutrient-rich (tryptic soy broth [TSB]) and nutrient-limited (Mueller–Hinton broth [MH]) media, with and without supplementation of 1% fetal bovine serum (tryptic soy broth supplemented with 1% FBS [TSB1] and Müller–Hinton supplemented with 1% FBS [MH1]), to mimic environmental and host-like conditions. Total RNA was sequenced using the Oxford Nanopore MinION platform, and gene expression was quantified using featureCounts and Salmon, followed by differential expression analysis with DESeq2. Results revealed that culture conditions significantly shaped transcriptomic profiles. TSB1 promoted the highest and most consistent expression of classical virulence genes such as *aerA*, *exeC*, and *fliP*, due to serum-derived host signals. In contrast, MH induced higher expression of genes linked to motility and early host interaction, including *flpI* and *exeB*, despite overall lower transcriptional activity. These findings highlight the complementary expression of virulence factors under distinct nutritional conditions. Heatmaps and principal component analysis (PCA) confirmed clustering of expression profiles across media types. In relation to our findings, TSB1 is therefore recommended as the primary medium for bacterin production in autogenous vaccine development. However, combining cultures grown in both TSB1 and MH may capture a broader antigen repertoire, enhancing immune recognition and protection. This transcriptomics-based strategy presents as a rational framework for designing next-generation autogenous vaccines in aquatic veterinary medicine.

## Introduction

The ongoing growth of global aquaculture is one of the cornerstones of the 2030 Agenda for Sustainable Development and offers a significant opportunity to address issues such as food security and malnutrition, particularly in developing countries [1]. In 2022, total aquaculture production reached an all-time high of 130.9 million tonnes, significantly surpassing the 92.3 million tonnes produced by the fisheries industry [2]. However, aquaculture growth may be undermined by the impact of diseases, which can rapidly devastate populations of farmed fish. The causes of this vulnerability are many, including high stocking densities, farming of non-native species, and monoculture practices. Furthermore, use of aquatic resources is affected by climate change, particularly related to added stress of thermal fluctuations and increased intensity and frequency of severe weather events reflected in hydrological regime extremes (droughts and floods).

The abovementioned factors and other risk factors support a situation where the threat of bacterial infections is higher than ever before [3–6]. In many cases, fish farmers have been forced to use large quantities of antibacterial substances to control disease outbreaks, which, in the long term, has contributed to the rise of antimicrobial resistance and the spread of resistance-related genes (ARGs) within bacterial populations [7, 8].

To address these challenges, scientific research is increasingly turning to molecular and computational approaches to better understand pathogen behavior and support targeted vaccine development.

Among bacteria that can be regarded as targets for development of autogenous vaccines in aquatic veterinary medicine, the pathogenic *Aeromonas* spp.—*Aeromonas salmonicida* subsp. *salmonicida*, *Aeromonas hydrophila*, or others (*A. sobria*, *A. veronii*) are among the most relevant bacterial pathogens in aquaculture [10, 11] and represent key targets for vaccine development. Among the many members of the Aeromonad cluster, *A. salmonicida* subsp. *salmonicida* and *A. hydrophila* species are most frequently associated with substantial economic losses owing to their pathogenicity, prevalence, and the severity of the diseases they cause across diverse farmed fish populations [12, 13]. *A. salmonicida* subsp. *salmonicida* is a Gram-negative, facultatively anaerobic, nonmotile, psychrophilic bacterium that thrives in cold-water environments. It is the etiological agent of furunculosis, a systemic and often fatal disease primarily affecting salmonids. The pathogen is capable of persisting in aquatic habitats and is readily transmitted through water, direct fish-to-fish contact, and contaminated equipment. Its virulence is mediated by several key factors, including extracellular proteases, hemolysins, and a surface A-layer protein, which facilitate host invasion and immune evasion [13]. Furunculosis manifests in acute and chronic forms. Acute cases are characterized by signs of septicemia, such as lethargy, anorexia, darkened pigmentation, and extensive hemorrhaging of the skin, fins, and internal organs. Chronic presentations typically include localized abscesses (furuncles), ulcerative skin lesions, exophthalmia, and splenomegaly [13, 14]. Both forms are associated with significant mortality, especially in intensive salmonid production systems. Similarly, *A. hydrophila* is also a Gram-negative, facultatively anaerobic, motile, but mesophilic bacterium commonly found in warm-water aquaculture environments. It is the principal causative agent of motile Aeromonas septicemia (MAS), a disease characterized by hemorrhagic septicemia, external ulceration, and internal organ damage. *A. hydrophila* has a broad host range and demonstrates notable strain variability in virulence, contributing to its widespread distribution and persistence in diverse aquatic systems [15, 16]. Transmission occurs through waterborne exposure, particularly under stressful conditions such as high temperatures, overcrowding, and poor water quality [16]. Given their epidemiological significance, both *A. salmonicida* and *A. hydrophila* warrant continued attention in their molecular characterization and vaccine development. Targeted interventions against these pathogens are essential to reduce disease incidence, improve fish health, and support the long-term sustainability of global aquaculture.

To overcome this threat, aquatic veterinarians and animal health experts recommend reducing the use of antibiotics in farming conditions and instead implementing rigorous biosecurity strategies, particularly vaccines. Among these, autogenous vaccines are noteworthy [17]. Developed from pathogens isolated directly from a specific fish population or aquaculture facility, they provide a highly targeted approach to disease prevention when commercial vaccines are unavailable, ineffective, or mismatched to circulating strains. Especially useful in managing localized outbreaks and emerging diseases, autogenous vaccines are tailored to the antigenic profile of the pathogens affecting the stock [17]. Recent advances in biotechnological tools—such as transcriptomic profiling and cultivation-dependent gene expression analysis—offer new opportunities to investigate pathogen virulence mechanisms and guide vaccine development [18]. Thanks to the accessibility and cost-effectiveness of sequencing platforms such as Oxford Nanopore Technologies (ONT), these approaches are now within reach of the broader research community. Building on these developments, we conducted a molecular investigation of clinical isolates of *Aeromonas salmonicida* ssp. *salmonicida* and *Aeromonas hydrophila*, integrating transcriptomic profiling and virulence gene expression analysis. Our aim was to identify cultivation-related differences that can inform the design of more effective, strain-specific autogenous vaccines and advance their utility in aquatic veterinary medicine.

## Materials and methods

### Sample collection

The investigated *A. salmonicida* subspecies *salmonicida* isolates were collected from symptomatic Alsatian char, brook trout (*Salvelinus alpinus* $\times$ *fontinalis*), Arctic char, and brown trout, during disease outbreaks presenting with clinical symptoms typical for furunculosis. The samples were collected from several aquaculture establishments in the region of Bavaria, Germany, between 2018 and 2020. Sampling locations were from the Muhlbach, Lauterach, Walchbach, Lech, Saussbach, Kolbersbach, and Grobenbach rivers. The fish samples were delivered to the Bavarian Animal Health Service, Fish Health Service division (Tiergesundheitsdienst Bayern e.V./Fischgesundheitsdienst, Poing, Germany) as a part of a routine laboratory diagnosis/investigation of clinical cases reported by fish farmers. In the case of *A. hydrophila* isolates, those were collected during the outbreaks taking place in Thailand from May to December 2016, and were previously described by Nicholson et al. (22). In the case of both pathogens, their taxonomical status was previously confirmed by molecular analysis [19, 20].

### Bacterial culture

Eight *Aeromonas salmonicida* subsp. *salmonicida* and two *Aeromonas hydrophila* isolates were stored in a cryotube system and reactivated on Columbia blood agar plates (Thermo Fisher Scientific, Wesel, Germany). Colonies were verified on the basis of morphology (round shape) and positive beta-hemolytic activity, and confirmed isolates were used for subsequent subcultures. Each isolate was then grown under four distinct liquid media conditions: nutrient-rich tryptic soy broth (TSB; Merck, Darmstadt, Germany) and nutrient-limited Mueller–Hinton broth (MH; Sigma-Aldrich, Darmstadt, Germany), each with or without supplementation of 1% fetal bovine serum (TSB1, MH1, respectively; Sigma-Aldrich, Darmstadt, Germany). This design yielded a total of 32 biological samples for *A. salmonicida* and 8 for *A. hydrophila*. Variation in the concentration and composition of nutrients in the culture media was expected to affect the expression levels of genes associated with bacterial virulence and motility mechanisms. In nutrient-limited conditions, where bacteria are likely to be actively seeking host signals or alternative nutrient sources, an upregulation of these genes is anticipated. To simulate the host environment more closely, fetal bovine serum (FBS) was added to select media. The inclusion of FBS served to mimic in vivo conditions by introducing host-derived factors such as proteins, lipids, and signaling molecules, thereby providing physiologically relevant stimuli that may influence bacterial gene expression. Culture conditions were optimized on the basis of preliminary growth curve analyses (Additional file 1), and all cultures were grown in shaking incubators. *Aeromonas salmonicida* subsp. *salmonicida* isolates were incubated at 20 $^{\circ }$C and consistently reached the late logarithmic phase after 30–34 h, which was reproducible across repeated experiments. This lower incubation temperature was chosen to better reflect natural infection conditions, since most salmonids cannot tolerate prolonged exposure to water temperatures above 20 $^{\circ }$C. In contrast, *A. hydrophila* isolates were incubated at 28 $^{\circ }$C and reached the late logarithmic phase after 20–24 h, consistent with their ecological background as strains primarily associated with warm-water aquaculture in Southern Asia.

### RNA isolation

RNA extraction was performed with Nucleozol (Macherey-Nagel, Dueren, Germany) according to the manufacturer’s protocols. The resulting concentrations of synthesized cDNA were then checked using a Qubit$^{\textrm{TM}}$ 4 Fluorometer (Thermo Fisher Scientific, Waltham, MA, USA) and were in the range of 55–60 ng/${\upmu }$L. In turn, the quality of the samples was checked using a Nanodrop 1000 Spectrophotometer (Thermo Fisher Scientific, USA). The absorbance parameters for the samples tested were 260/230: 1.7–2.2 and 260/280 1.7–2.3.

### cDNA synthesis

In the cDNA synthesis step, about 1 ug of total RNA from each sample was used for the reaction along with the iScript cDNA Synthesis Kit (Bio-Rad), following the manufacturer’s protocol. We incubated the complete reaction mix in the thermal cycler for 5 min at 25 $^{\circ }$C for priming, 20 min at 46 $^{\circ }$C for reversing transcription, and 1 min at 95 $^{\circ }$C for reverse transcription (RT) inactivation. The final cDNA sample concentration was detected again on the Qubit$^{\textrm{TM}}$ Fluorometer and was in the range of 60–100 ng/${\upmu }$L. All cDNA samples were kept at 4 $^{\circ }$C until library preparation.

### Library preparation and transcriptome data analysis

Next, cDNA was sequenced using the Oxford Nanopore MinION platform with the Native Barcoding Kit version 24 and a FLO-MIN114 (R10.4.1) flow cell. Basecalling, demultiplexing, adapter trimming (Porechop version 0.2.4) [21], and quality assessment (FastQC version 0.11.9) [22] were performed accordingly. Gene expression was quantified using featureCounts version2.0.3 [23] on the basis of annotated genes from the reference genomes:

- *Aeromonas salmonicida* subsp. *salmonicida* A449 (NC_009348.1)
- *Aeromonas hydrophila* strain CSUSB2 (CP083944.1)

For virulence gene analysis, a tblastn search was conducted with custom protein queries, and transcript-level quantification was performed using Salmon version 1.10.2 [24]. All bioinformatic steps were conducted in the usegalaxy.eu environment.

Differential expression analysis was performed in R (version 4.3.0) [25] using the DESeq2 version 1.40.2 [26] package. Genes were considered differentially expressed if they met the thresholds of adjusted *p*-value < 0.05 and |log FoldChange| > 1. Data visualization was performed with ggplot2 [27], ComplexHeatmap [28], and ggforce [29].

## Results

**Figure 1:**
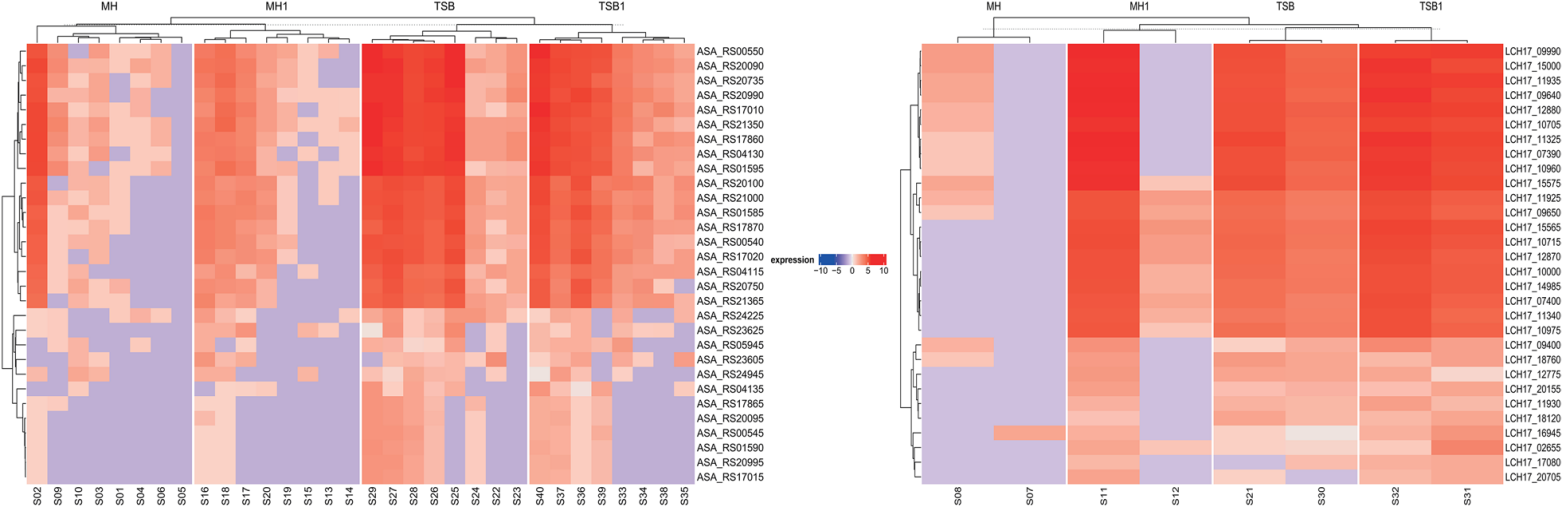
**Heatmap of the top 30 differentially expressed genes in (A)** ***Aeromonas salmonicida*** **subsp.** ***salmonicida*** **and** **(B)** ***Aeromonas hydrophila***. Rows correspond to genes (locus tags ASA_RSXXXXX or LCH17_XXXXX) and columns to biological replicates (S codes) grouped by culture condition. Culture conditions: *MH* Mueller–Hinton, *MH1* Mueller–Hinton + 1% FBS, *TSB* Tryptic Soy Broth, *TSB1* Tryptic Soy Broth + 1% FBS.

Sequencing produced long reads, with an average read length of 610 nt, a median of 299 nt, and a maximum read length exceeding 477 kb (Supplementary File: Seq_data). The sequence data obtained in this study were deposited in the Sequence Read Archive (SRA) of the National Center for Biotechnology Information (NCBI) under accession no. PRJNA1282914. In the case of *Aeromonas salmonicida* ssp. *salmonicida*, with isolates cultured under four distinct experimental conditions, we observed that isolates grown in tryptic soy broth (TSB) and tryptic soy broth supplemented with 1% FBS (TSB1) media exhibited an overall higher expression level across numerous loci compared with those cultured in Mueller–Hinton broth (MH) and Mueller–Hinton broth supplemented with 1% FBS (MH1). This trend indicates a culture media-related shift in transcriptional activity, although the biological roles of many of these loci remain uncharacterized. Importantly, several of the represented loci correspond to ribosomal RNA (rRNA) genes, which, while not encoding proteins, play a crucial role in the process of protein synthesis by forming the structural and functional core of ribosomes. No significant differences in gene expression were observed between the MH and MH1 or TSB and TSB1 medium pairs, indicating consistent transcriptional profiles within each respective culture condition (Figure 1).

**Figure 2:**
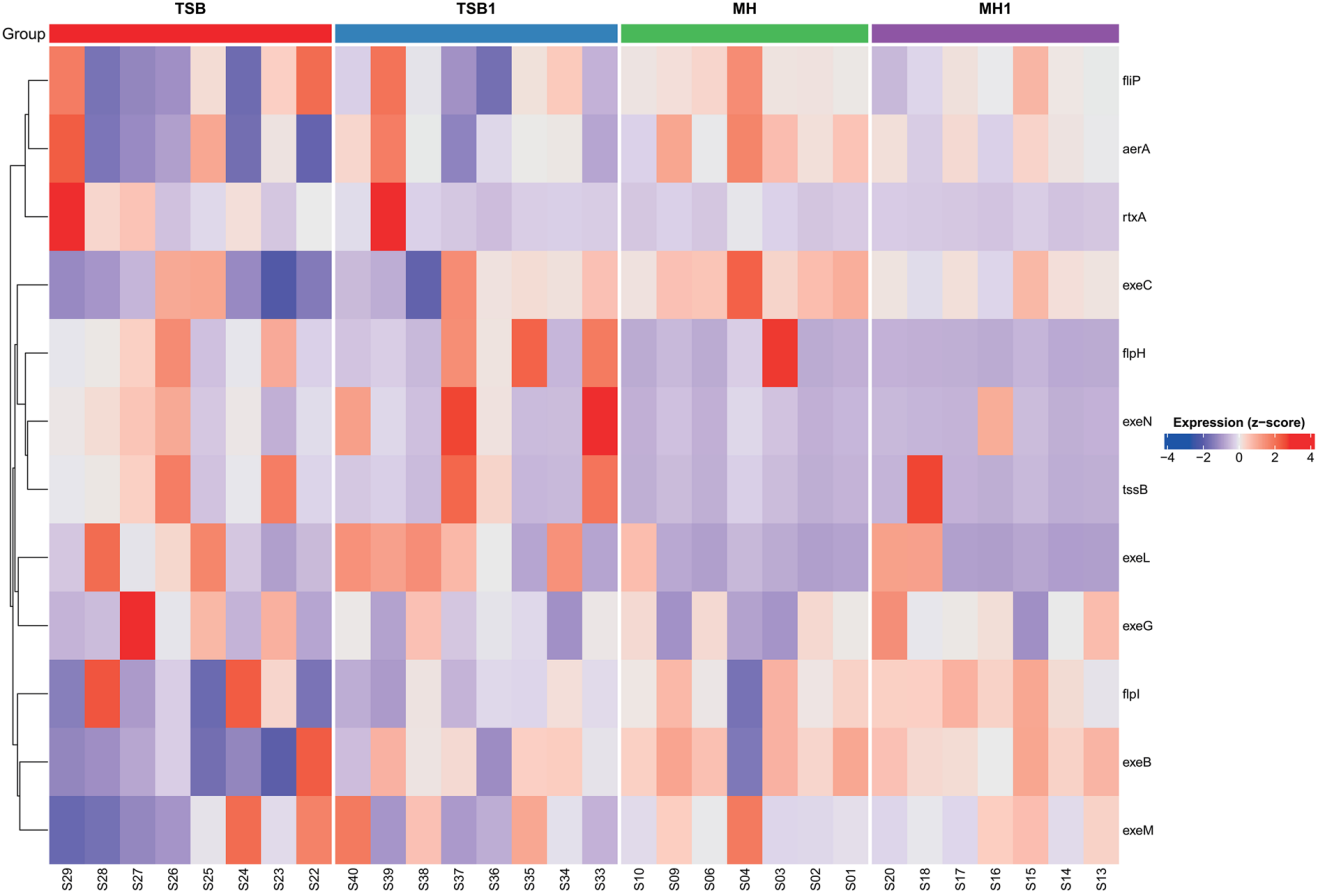
**Heatmap of virulence genes in** ***Aeromonas salmonicida*** **subsp.** ***salmonicida***. Rows correspond to virulence genes, and columns to biological replicates (S codes) grouped by culture condition. Culture conditions: *MH* Mueller–Hinton, *MH1* Mueller–Hinton + 1% FBS, *TSB* Tryptic Soy Broth, *TSB1* Tryptic Soy Broth + 1% FBS.

To further evaluate the trend identified in *A. salmonicida* isolates, two samples of *A. hydrophila*—associated with disease outbreaks—were included in the analysis as a comparative reference group. Given their limited number and distinct origin, these samples were not used as a basis for drawing statistically significant conclusions but rather served to qualitatively assess the broader gene expression patterns (Figures 1 and 2).

Interestingly, a similar tendency was observed for the *A. hydrophila* isolates, which, despite their outlier status, also exhibited overall higher transcriptional activity when cultured in TSB compared with MH. Multiple loci showed differential expression, reflecting the pattern seen in *A. salmonicida*. This consistency across species supports the notion that nutrient-rich media such as TSB may have a notable impact on global transcriptional activity.

In addition, we analyzed the expression patterns of selected virulence genes (*fliP*, *aerA*, *rtxA*, *exeC*, *flpH*, *exeN*, *tssB*, *exeL*, *exeG*, *flpI*, *exeB*, and *exeH*) (Figure 2). The results revealed trends consistent with the overall transcriptomic profiles. In *Aeromonas salmonicida* subsp. *salmonicida*, the TSB and TSB1 groups exhibited the highest overall expression levels across the evaluated virulence loci. However, in the TSB group, the downregulation of gene expression was more widespread, with multiple virulence genes showing reduced expression across several samples. In contrast, the TSB1 group demonstrated a more consistent expression pattern, characterized by fewer downregulated genes and predominantly neutral or upregulated expression levels. In addition, serum supplementation in TSB1 was associated with a more stable and uniform activation of virulence-associated genes.

**Figure 3:**
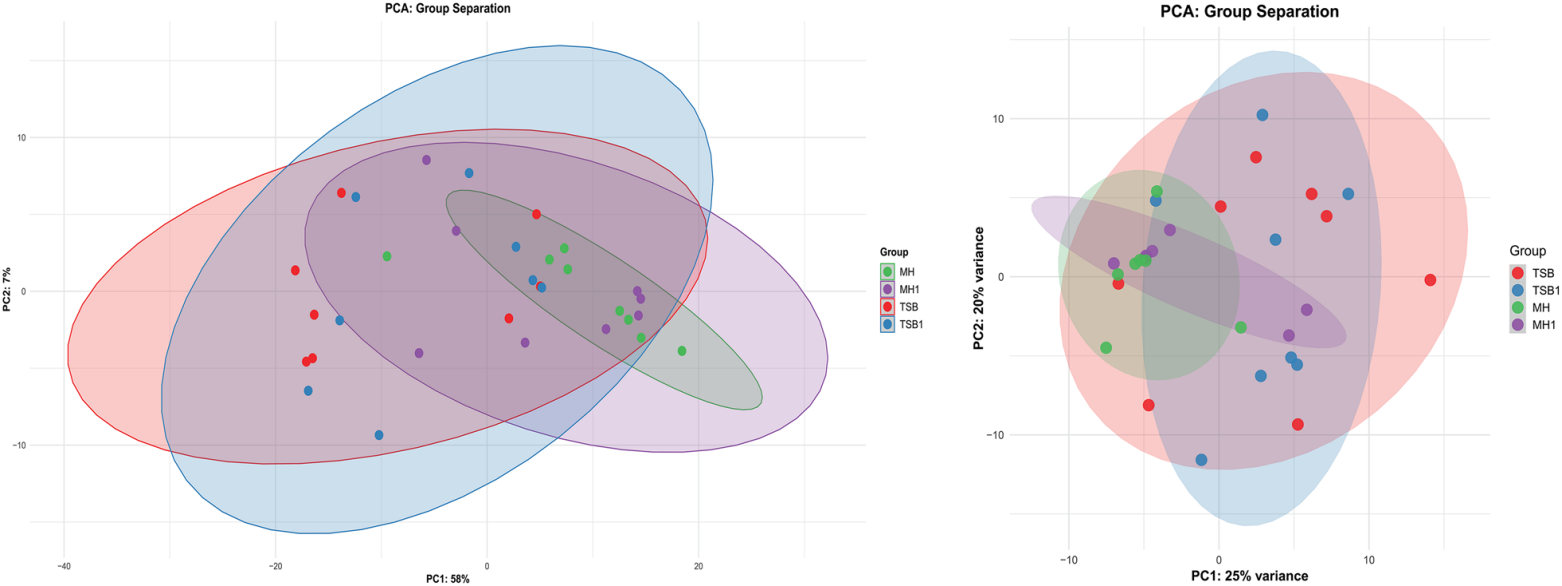
**Principal component analysis (PCA) of** ***Aeromonas salmonicida*** **subsp.** ***salmonicida***. (**A**) PCA based on expression data from all detected genes. (**B**) PCA based on expression data limited to virulence-associated genes. Each point represents a biological replicate (S codes), grouped by culture condition. Culture conditions: *MH* Mueller–Hinton, *MH1* Mueller–Hinton supplemented with 1% FBS, *TSB* Tryptic Soy Broth, *TSB1* Tryptic Soy Broth supplemented with 1% FBS.

PCA was conducted to reduce the dimensionality of the dataset and visualize the main axes of variation across the defined experimental groups: MH, MH1, TSB, and TSB1. Each point represents an individual sample, while ellipses denote the 95% confidence interval for each group, based on the multivariate distribution of the first two principal components. In the first PCA figure (Figure 3B), the first two principal components accounted for 46% of the total variance (PC1 = 25%, PC2 = 21%). A moderate level of group separation is observed, particularly between the TSB and MH clusters. However, substantial overlap remains among all four groups, suggesting shared underlying variance structures or partially overlapping phenotypic or molecular profiles. The elliptical distributions indicate that group means are distinguishable but not clearly segregated in the first two principal dimensions. In the second PCA figure (Figure 3B), PC1 explained a substantially higher portion of the total variance (58%), whereas PC2 captured a relatively minor proportion (7%). In this configuration, improved group separation is evident along the PC1 axis. Notably, MH and TSB1 groups show greater spatial distinction, indicating that the main driver of variation (as captured by PC1) contributes meaningfully to group differentiation. Despite improved separation, some degree of overlap persists, particularly between MH1 and TSB, suggesting partial similarity in their profiles. The tighter clustering observed for certain groups (e.g., TSB1) may reflect higher within-group homogeneity.

**Figure 4:**
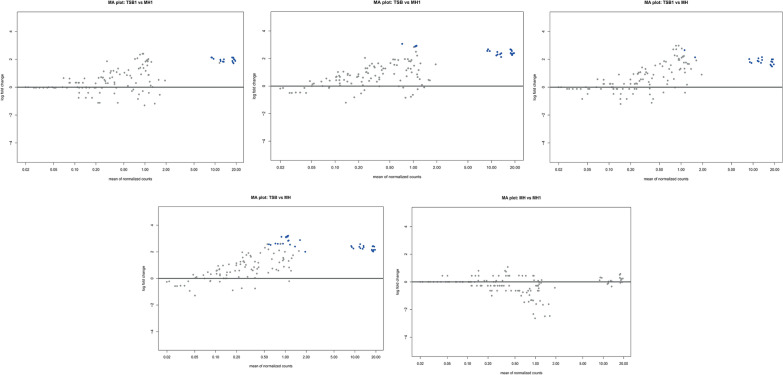
**MA plots of** ***Aeromonas salmonicida*** **subsp.** ***salmonicida*** **gene expression.** Each panel shows log fold change (*y*-axis) versus mean of normalized counts (*x*-axis) for pairwise comparisons between culture conditions. Gray dots represent genes without significant change, and blue dots indicate differentially expressed genes. Culture conditions: *MH* Mueller–Hinton, *MH1* Mueller–Hinton supplemented with 1% FBS, *TSB* tryptic soy broth, *TSB1* tryptic soy broth supplemented with 1% FBS.

**Figure 5:**
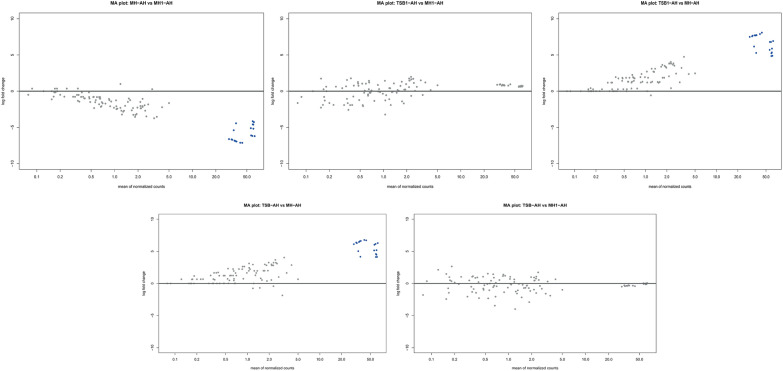
**MA (mean-average plot) plots of** ***Aeromonas hydrophila*** **gene expression.** Each panel shows log fold change (*y*-axis) versus mean of normalized counts (*x*-axis) for pairwise comparisons between culture conditions. Gray dots represent genes without significant change, and blue dots indicate differentially expressed genes. Culture conditions: *MH* Mueller–Hinton, *MH1* Mueller–Hinton supplemented with 1% FBS, *TSB* tryptic soy broth, *TSB1* tryptic soy broth supplemented with 1% FBS.

Differential gene expression analysis was performed using the DESeq2 package for five pairwise comparisons between the experimental groups: TSB, TSB1, MH, and MH1. For each comparison, MA plots were generated (log_2_ fold change versus mean expression level, *p* > 0.05), both for *Aeromonas salmonicida* subsp. s*almonicida* (Figure 4) and *Aeromonas hydrophila* (Figure 5). The largest differences in expression for *Aeromonas salmonicida* subsp. *salmonicida* were observed in the TSB versus MH and TSB versus MH1 comparisons. In both cases, a distinct cluster of genes showed significantly increased expression in TSB samples, including numerous genes with relatively high expression levels (high baseMean). This indicates strong transcriptomic regulation under the conditions represented by TSB. A similar, although slightly less pronounced, pattern was observed in the TSB1 versus MH comparison, where a subset of genes was also upregulated in the TSB1 condition. In contrast, the TSB1 versus MH1 comparison revealed a substantially lower number of differentially expressed genes. No significant differences were identified in the MH versus MH1 comparison, indicating a highly similar gene expression profile between these two groups. In summary, the analysis indicates a strong effect of the conditions represented by TSB and TSB1 on gene expression, particularly in comparison to MH and MH1. Comparisons involving the MH group showed the highest degree of transcriptomic divergence, whereas MH and MH1 appear to be nearly indistinguishable in terms of global gene expression.

Similar patterns were observed for *Aeromonas hydrophila*, where the strongest expression differences occurred between TSB-AH (AH-affix for *Aeromonas hydrophila*) and MH-AH, as well as between TSB1-AH and MH-AH. In both cases, a number of genes exhibited significantly increased expression under TSB-AH and TSB1-AH conditions. These genes showed high mean expression values, suggesting that the observed changes may have biological relevance. Notably, the differences in both comparisons were centered around increased expression, with significantly positive log_2_ fold change values. In contrast, no statistically significant changes in gene expression were observed in the comparisons TSB1-AH versus MH1-AH and TSB-AH versus MH1-AH. This suggests that the transcriptomic profile of MH1-AH is more similar to that of TSB-AH and TSB1-AH than to MH-AH, potentially reflecting the influence of an additional factor associated with MH1 (e.g., prior exposure, altered physiological condition, or biological status). Finally, the comparison of MH-AH versus MH1-AH revealed only significantly downregulated genes in the MH1-AH group (negative log_2_ fold change), suggesting a marked repression of a specific subset of genes in this group. Unlike the other comparisons, all significant genes in this case showed decreased expression, which may indicate active suppression of specific biological processes in MH1-AH.

**Figure 6:**
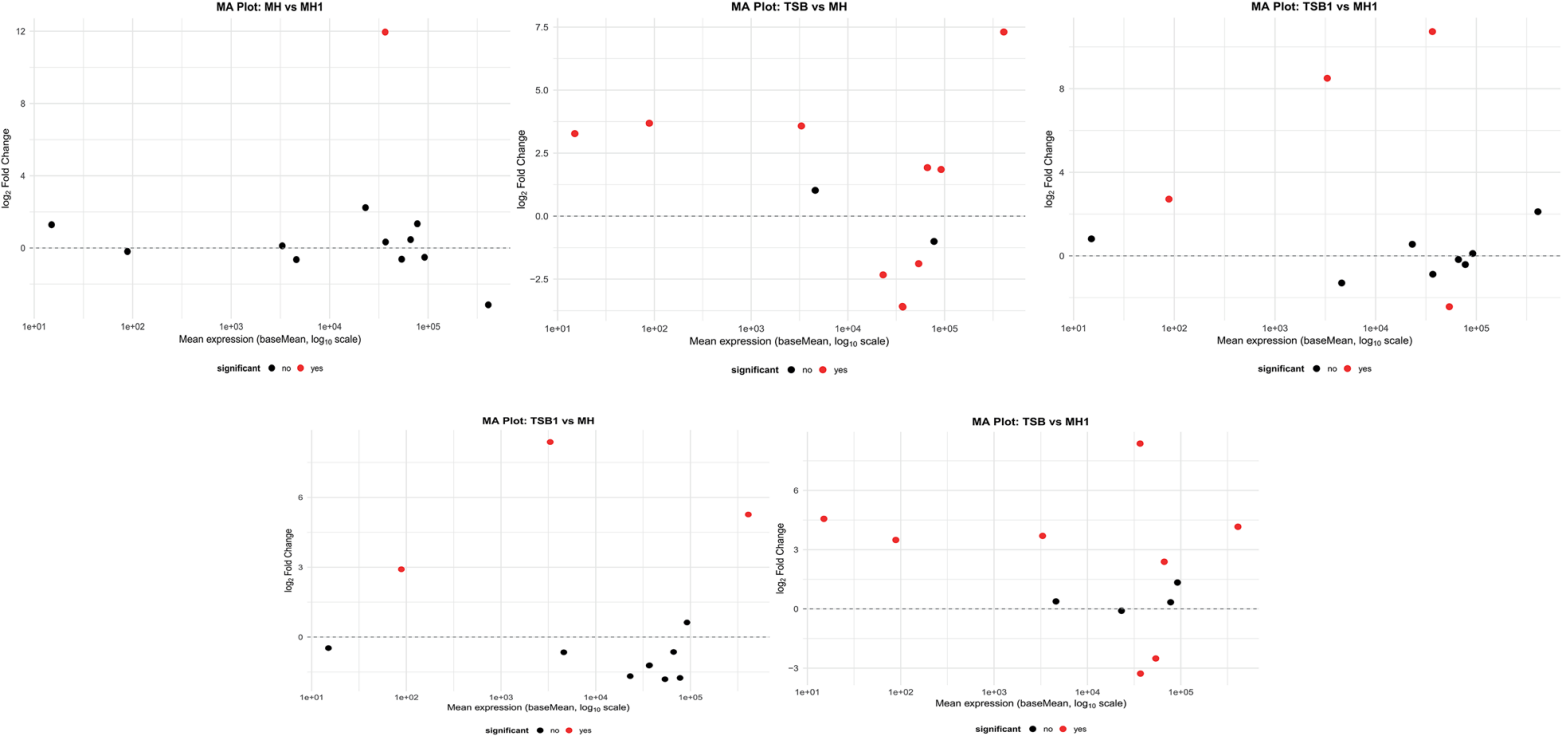
**MA plots of** ***Aeromonas salmonicida*** **subsp.** ***salmonicida*** **virulence genes.** Each panel shows log fold change (*y*-axis) versus mean expression (*x*-axis, baseMean in log, scale) for pairwise comparisons between culture conditions. Gray dots represent genes without significant changes, and red dots indicate significantly differentially expressed virulence genes. Culture conditions: *MH* Mueller–Hinton, *MH1* Mueller–Hinton supplemented with 1% FBS, *TSB* tryptic soy broth, *TSB1* tryptic soy broth supplemented with 1% FBS.

In the analysis of virulence gene expression (Figure 6), a comparison between TSB and MH revealed upregulation of two genes (*aerA* and *fliP*) in the TSB group, with log_2_ fold change values exceeding three. These genes belonged to the group of low to moderately expressed transcripts. In the MH versus MH1 comparison, a single gene (*flpI*) was found to be significantly upregulated in MH1, with a log_2_ fold change greater than 10. This indicates strong activation of flpI despite the overall minor differences between MH and MH1 observed in the global expression analysis. In contrast, the TSB versus MH1 comparison showed the highest number of differentially expressed virulence genes. Among them, both strongly upregulated and downregulated genes were identified in the TSB group. Some of these genes, such as *aerA* and *fliP* (upregulated), and *exeG* and *exeM* (downregulated), exhibited high baseMean values, suggesting that the observed differences may be biologically relevant. These results point to a strong contrast in the virulence gene expression profiles between TSB and MH1.

The comparison of TSB1 versus MH also revealed significant differences, with the majority of differentially expressed genes showing a positive log_2_ fold change, indicating higher expression in TSB1. The identified genes included transcripts that were both lowly and highly expressed, suggesting a broad range of transcriptional changes in this group. Finally, in the TSB1 versus MH1 comparison, several genes (*aerA*, *fliP*, *flpI*, *exeC*, etc.) were found to be significantly differentially expressed, including both upregulated and downregulated transcripts in the TSB1 group. The range of log_2_ fold change values was variable, and some of the significant genes showed high mean expression levels.

## Discussion

The selection of culture media in this study was guided by the objective to explore how different culture environment conditions influence virulence gene expression. TSB and MH were chosen to represent nutrient-rich and nutrient-limited environments, respectively [19]. This contrast allowed us to capture a broader spectrum of gene expression patterns, as bacteria often activate different sets of genes depending on the availability of nutrients and environmental stressors. Rich media such as TSB typically promote rapid growth and the expression of classic virulence factors [30], whereas low-nutrient conditions such as MH can trigger the expression of genes associated with stress response, motility, adhesion, and host interaction [31]. To further mimic host-like conditions, both media were supplemented with 1% fetal bovine serum (FBS), resulting in the TSB1 and MH1 variants. The addition of FBS was intended to simulate the in vivo environment by introducing host-derived signals and components, potentially enhancing the ecological relevance of the observed expression profiles [32]. This experimental design allowed for the identification of both constitutively and conditionally expressed virulence factors, providing a comprehensive vaccine antigen expression. The transcriptomic analysis of *Aeromonas salmonicida* subsp. *salmonicida* isolates revealed clear differences in virulence-associated gene expression depending on the cultivation medium. Among the tested conditions, TSB1 exhibited the highest overall expression levels, likely due to the presence of serum components that better mimic host-like environments. Meanwhile, TSB also promoted the expression of several key virulence genes, although it was accompanied by notable downregulation of others—specifically, *exeG*, *flpI*, *exeB*, and *exeM*. This highlights the complexity of the bacterial transcriptional response and suggests that individual virulence loci may be subject to divergent regulation depending on subtle environmental cues [33].

One of the most prominent virulence markers, the *aerA* gene, encodes a pore-forming hemolysin that is widely recognized for its role in pathogenesis. Prior studies have demonstrated a significant reduction in bacterial lethality following deletion of aerA, reinforcing its critical contribution to virulence [34, 35]. In addition, multiple genes involved in type II secretion systems (T2SS), such as *exeB*, *exeC*, and *exeM*, were strongly expressed in nutrient-rich, serum-supplemented environments. These systems are essential for secreting a variety of exotoxins, further underlining their importance in bacterial pathogenicity. Another notable gene, *fliP*, encodes a protein integral to the flagellar type III secretion export apparatus, which is fundamental for flagellar assembly and motility [36]. Its robust expression under TSB1 conditions suggests that serum components may upregulate genes involved in both structural assembly and pathogenesis. Similar patterns of increased virulence gene expression in serum-enriched media have been reported in other bacterial species, such as *Staphylococcus aureus*, further supporting this interpretation [37].

Interestingly, the *tssB* gene, encoding a sheath protein of the type VI secretion system (T6SS), was significantly expressed in both TSB and TSB1. However, since T6SS-related virulence is typically limited to a subset of highly virulent *Aeromonas* strains [38], its utility as a universal virulence marker remains uncertain. In contrast to nutrient-rich conditions, MH and MH1 generally supported lower global transcriptional activity, aligning with expectations for nutrient-limited environments. Nevertheless, certain virulence genes—most notably *flpI*, *exeB*, and *flpH*—were more strongly expressed under MH conditions. These genes are associated with motility, adhesion, and host interaction, as they form part of the type IVb pili system (*flpI*, *flpH*) and T2SS machinery (*exeB*) [39]. This pattern suggests that under nutrient scarcity, *Aeromonas* may shift toward a physiological state favoring environmental exploration and early colonization mechanisms. This observation is particularly relevant in the context of the pathogen’s natural ecology. In aquatic habitats, nutrient limitation is common, and successful infection may rely on the bacteria’s ability to locate and colonize host tissues [40, 41]. Thus, the upregulation of dual-function genes under MH conditions likely represents an adaptive response linking survival and pathogenesis—an insight with direct implications for vaccine development.

Notably, while MA plots provided a useful summary of statistically significant gene expression changes, heatmap visualizations revealed additional biologically relevant patterns. In particular, the coordinated shifts in expression across the full panel of virulence-associated genes, including patterns of distributed repression or coexpression, were more apparent in heatmaps. This underscores the importance of visual analytical tools in complementing traditional statistical outputs. Taken together, these findings suggest that TSB and TSB1 media are favorable for inducing the expression of classical virulence factors and may serve as suitable conditions for producing highly immunogenic bacterins for autogenous vaccines. However, MH-based cultivation may induce complementary virulence determinants related to motility and host engagement, which are often underrepresented in nutrient-rich media. This highlights the critical role of cultivation conditions in shaping the transcriptomic and immunogenic landscape of bacterial vaccine candidates. The inclusion of 1% FBS in TSB was designed to simulate host-associated conditions more closely, thereby enhancing the ecological and clinical relevance of the expressed antigens. The observed increase in overall expression levels under TSB1 conditions supports this rationale and reinforces its value as a basis for vaccine development.

While our transcriptomic analysis provides valuable insights into the expression dynamics of virulence-associated genes under different culture conditions, this study did not include functional immunogenicity testing to validate the protective efficacy of the identified antigen profiles. As such, the conclusions regarding vaccine potential are based on gene expression patterns rather than direct immunological outcomes. Future work should incorporate in vivo vaccination trials and immunological assays to confirm the antigenicity and protective value of bacterins produced under optimized cultivation conditions, as well as host response in terms building up of immunity.

In light of these insights, we propose a multimodal cultivation strategy for autogenous vaccine production. By combining bacterins from the same isolate grown under both nutrient-rich (TSB1) and nutrient-limited (MH) conditions, it may be possible to capture a broader and more representative spectrum of virulence-associated antigens. Such dual-condition formulations could mimic distinct transcriptional states associated with colonization and invasion, potentially leading to more robust and comprehensive immune responses. Future research should explore this approach further, incorporating diverse *Aeromonas* strains, variable culture conditions, and immunological efficacy testing in vivo, ultimately guiding the design of more effective and ecologically relevant vaccines.

## Conclusions

Among all tested conditions, tryptic soy broth with 1% fetal bovine serum (TSB1) consistently promoted the highest and most diverse expression of key virulence genes, making it the most suitable single-condition choice for producing bacterins in autogenous vaccines. While TSB1 alone offers a strong foundation for immunogenic antigen profiles, incorporating a second component cultured under alternative conditions (such as MH) may capture complementary transcriptional states, particularly those relevant to motility/mobility and early interactions with the host. This supports the development of complex, two-component vaccine formulations that better reflect the pathogen’s full virulence potential. TSB1-grown isolates are suitable for single-component vaccine production, while dual-condition strategies may further enhance vaccine coverage and efficacy. These findings provide transcriptomics-based rationale for optimizing bacterial culture protocols in next-generation autogenous vaccine design.

## Additional file

**Additional file 1** Growth curve of *Aeromonas hydrophila* (28 °C, TSB) and Aeromonassalmonicida subsp. salmonicida (20 °C, TSB)-Semi-logarithmicplot showing OD and log CFU/mL values during growth.

## Data Availability

Accession no. PRJNA1282914.
