# Supplementary figures and images for: Transcriptomic insights into cultivation-driven virulence in Aeromonas spp.: a new approach to optimizing autogenous vaccines in aquatic veterinary medicine
Source: Vet Res. 2026 Jan 24;57:16. doi: 10.1186/s13567-025-01692-9 (PMC12833933; doi:10.1186/s13567-025-01692-9)

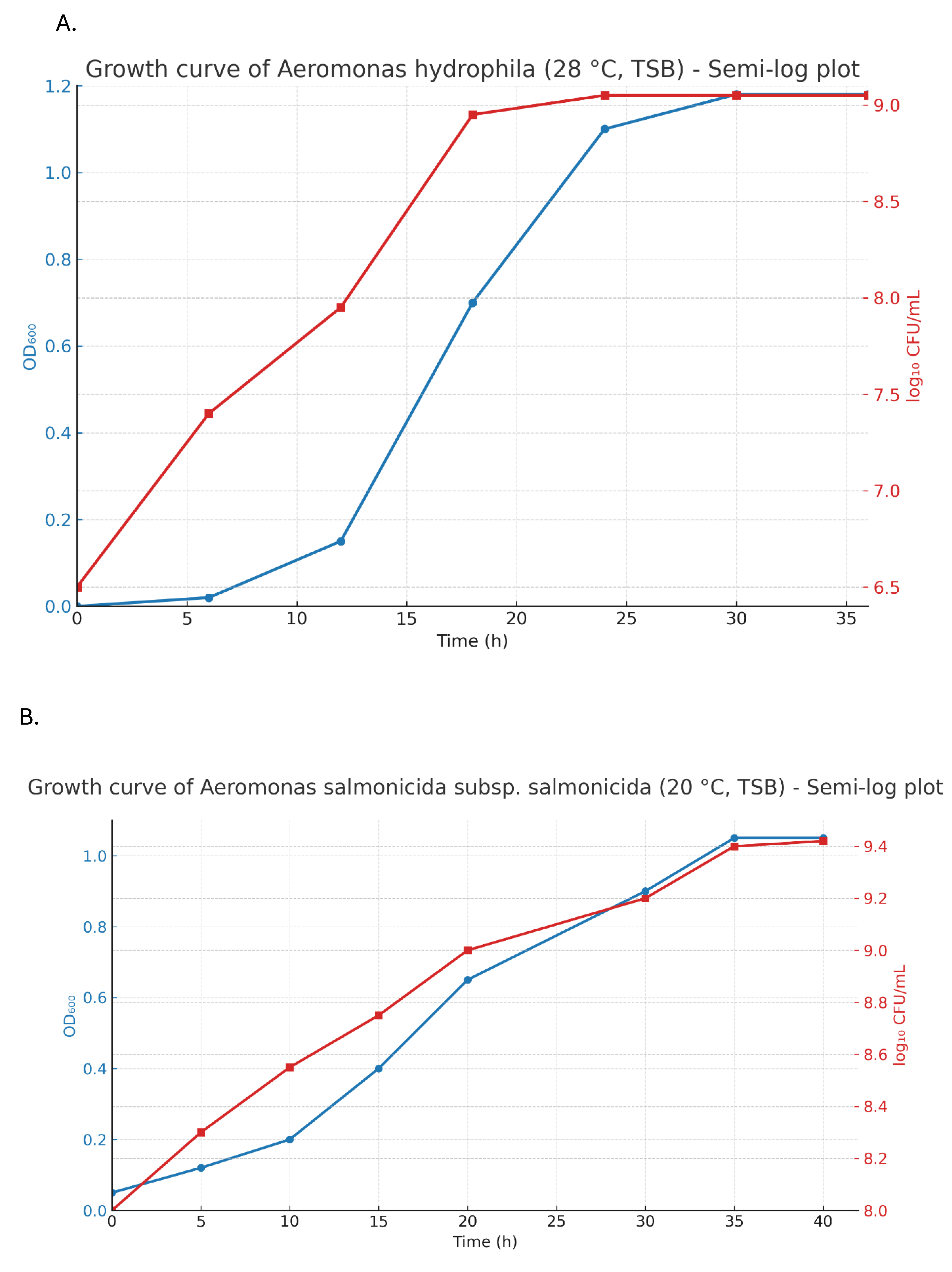

Supplement: Supplementary file 1 — Additional file 1 Growth curve of Aeromonas hydrophila (28 °C, TSB) and Aeromonassalmonicida subsp. salmonicida (20 °C, TSB)-Semi-logarithmicplot showing OD and log CFU/mL values during growth. [file 13567_2025_1692_MOESM1_ESM.tif]
